# Narratives on Sex and Contraception From Pregnant Adolescent Women in a Northern Province in Thailand: A Phenomenological Study

**DOI:** 10.1177/00469580211056219

**Published:** 2021-12-09

**Authors:** Panitsara Leekuan, Ros Kane, Panpimol Sukwong

**Affiliations:** 1 School of Nursing, 90440 University of Phayao, Phayao, Thailand; 2 School of Health and Social Care, 4547 University of Lincoln, Lincoln, UK; 3 School of Nursing, 90440 University of Phayao, Phayao, Thailand

**Keywords:** adolescent pregnancy, narration, sexual behaviour, contraception, phenomenology

## Abstract

Gaps in understanding, a lack of awareness of contraceptive use and a lack of control, related to gender dynamics, may influence the demand for contraception among adolescents and their decision-making around pregnancy prevention. This study explored the experiences of pregnant adolescents at the time of pregnancy and prior to becoming pregnant, examining attitudes toward sex and contraception. An interpretive phenomenological study guided by Heideggerian philosophy, analysed data from 30 in-depth interviews conducted with purposively selected consenting pregnant adolescents aged 15–19. Interviews were audio-recorded and transcribed verbatim and were analysed using a modified interpretative phenomenological approach. Participants exposed 5 key findings or experiences associated with sex and contraception: ‘*Premarital cohabitation and sex*’, ‘*Staying in the relationship*’, ‘*Unforeseen future*’, ‘*Parental conformity*’, and ‘*Male command*’. These findings can have far-reaching implications for the holistic understanding of the needs of adolescents in Thailand. They can be used to inform the development of appropriate and responsive interventions to support female and male adolescents, their families, and society. This includes interventions around reproductive health rights and sex education from health care providers, educators providing counselling to facilitate adolescents’ decision-making in order to reduce unintended adolescent pregnancy.

## What is Already Known About the Topic?

(1) Gender roles and power dynamics contribute to decision makeing around contraceptive use and need to be considered in the design of family planning strategies to enable informed decisions and to effectively increase contraceptive use among adolescents.
(2) The association between parental approval and adolescent sexual behaviour is crucial in engaging in cohabiting between sexual partners.

## How Does the Research Contribute to the Field?

(1) In this phenomenological study of pregnant adolescents, pregnancy frequently led to lack of several opportunities in further education and career progression.
(2) There is a need for developing skills in communication and negotiation around reproductive health issues prior to having sexual relationships and this offers not only new challenges but also fresh opportunities for sexual health promotion.

## What are the Research’s Implications Towards Theory, Practice, or Policy?

(1) This paper highlights the ways in which providing knowledge about contraception can improve the understanding of contraceptive methods. The uptake and utilisation of contraceptive methods is an important key to decreasing unintended/unplanned pregnancy rates. This study suggests that the policy makers should integrate sexual and reproductive health services into a one-stop convenient service, including the provision of an accessible and supportive family planning service for both male and female youth.
(2) This study offered various recommendations for health care providers. It also is a lens for health care providers to look more clearly in order to improve understanding of the participants’ experiences of being pregnant and to tailor their care particularly to their desires in terms of adolescent reproductive health care.

### Background

Pregnancy during adolescence in modern societies has a significant impact on adolescents and their babies, families and communities, with significant economic social, health and cultural implications.^1^ Adolescents are in a period of transition from childhood to adulthood and face profound physiological and psychological changes and challenges. Adolescents explore the world around them, and many engage in sexual relationships, placing them at risk of sexually transmitted infections (STIs) and unplanned and potentially unwanted pregnancy. Early sexual activity places adolescents at increased risk of adolescent pregnancy,^2^ particularly those who have unmet needs for, or reasons not to use contraception. It is recognised that sexual relationships commonly result in pregnancy between adult couples, but for adolescents this is an initial entrance to the world of adulthood and potentially motherhood. Adolescents face several barriers to positive reproductive and sexual health, including limited contraceptive access, unmet information needs and lack of negotiating power, which can put them at risk.^3^

Internationally, from an estimated 300 million female adolescents worldwide, approximately 21 million give birth every year.^4^ A report by the Thai Ministry of Public Health showed that adolescent births decreased from 51.1 per 1000 in 2013 to 31.3 per 1000 in 2019. However, this is still higher than the national goal of less than 25 per 1000.^5^ The large proportion of pregnancies in adolescent women in Thailand are unplanned and pose a risk of exposure to unsafe abortion.^6^ In Thailand, the abortion law is restrictive except in cases of risk to the woman’s physical or mental health, a high risk of genetic disease in the foetus, or rape or other sexual crimes.^7^ The penalty for performing abortions which do not meet these criteria is up to 5 years in prison or a fine of up to 10 000 Baht. As a result, illegal clinics and ‘back street’ abortions are available and widely used.

As using contraception is positively associated with family planning: inconsistent or lack of contraceptive use inherently increases the likelihood of pregnancy.^8^ Therefore, adolescent pregnancy essentially stems from a proportion of sexually active adolescents not using contraception consistently and effectively, and a rise in the rate of sexual activity in adolescence. In Thailand, the proportion of condom use during sexual debut increased from 65.0% in 2014 to 77.6% in 2019 among male adolescents, and from 64.2% to 80.0% among female adolescents. It is noticeable that increased contraception use was commensurate with a decline in adolescent pregnancy in Thailand,^6,9^ but Thai adolescents continue to engage in sexual activities in their mid-teens. The mean age of sexual debut in male adolescents is 15.2 years, and 15.4 years among females. Barriers to condom use for both genders include shame, discomfort, disapproval of using condoms, and faith in the partner’s honesty and fidelity.^5^

A survey of the National Statistical Office of Thailand showed that 88% of married women (aged of 20–24) and 80% of adolescents (15-19 years) were satisfied with modern contraceptive methods.^10^ The percentage of girls aged 15–19 years who were using (or whose partner was using) a contraceptive method was about 74%, but a proportion (17%) of these girls still had unmet need for contraception. A study by Yau, Adamu, Wongsawat and Songthap^11^ found that 75.8% of secondary school girls used contraception: 84% of young people had consistently used condoms, and only 10% used emergency pills. It is possibly that, in the current era of technological advancement, easy access to the internet allows swiftaccess to high quality, accurate information to inform sexual decision-making. Ready access to information and regular and consistent use of contraception potentially enables adolescents and their partners to exercise their rights to decide freely the number and spacing their children and to have the information, education and means to prevent pregnancy and STIs.

Adolescent sexual and reproductive health (ASRH) is a global public health concern as levels of sexual activity amongst adolescents have been on the increase in many countries worldwide. However, some adolescents are still faced with access barriers in relation to accurate information about their health and rights and are often uninformed about protection from pregnancies and sexually transmitted infections (STIs).^4^ Adolescent SRH care should therefore, include the provision of access to comprehensive sexuality education, with services to prevent, diagnose, and treat STIs, and counselling on family planning.^4^ It also plays a crucial role in empowering young people to know and exercise their rights, including the right to delay marriage and the right to refuse unwanted sexual advances.

Despite rapid changes in modern society, traditional gender norms remain pervasive in Thailand, conceptualising females as nurturing and obedient, and males as providers with authority over females. As a result, male power and dominance in decision-making, roles and responsibilities directly affect sexual and reproductive health and health-seeking behaviour. Culturally defined views of females render them less empowered to overtly use contraception when males are averse to family planning. Power manifested in contraceptive negotiations often includes the male partner taking a stance against using contraception, particularly condoms.^12^ Prominent reasons for male opposition to contraception use within a heterosexual union include the negative percention that is may lead to female promiscuity and lack of adequate knowledge about contraceptives.^13^ Inequitable gender norms result in male control over partners, and females not having full autonomy to make reproductive decisions, potentially resulting in unintended pregnancy and sexually transmitted infections (STIs).^14^

In response to the above, Thai authorities have tried to adopt various measures to prevent and reduce the incidence of adolescent pregnancy, including enacting the Prevention and Solution of the Adolescent Pregnancy Problem Act, B.E. 2559 (2016), to promote accessibility to reproductive health rights among adolescents.^15^ Nevertheless, the adolescent pregnancy rate remains high.

Few studies have examined pregnant adolescents’ experiences at the time of pregnancy and prior to becoming pregnant. Typically, at this age, attitudes toward sex and contraception are still developing. Consequently, this hermeneutic phenomenological study of pregnant adolescents’ experiences in Northern Thailand aims to improve understanding about the meaning and determinants of their sexual and contraceptive practices.

## Methods

### Study Setting

The pregnant adolescents were recruited from 3 hospitals in 3 districts within Northern Thailand which has the highest rate of adolescent pregnancy in the country and where the rate has been increasing continuously since 2010.

### Study Design

This was a phenomenological study examining the experiences of pregnant adolescents. The lead researcher contacted the directors of the 3 hospitals in order to provide information about the study and explain the purpose, procedure, inclusion and exclusion criteria for participants as well as the time period for data collection.

The following strategies were applied to approach participants. The lead researcher contacted nurses to introduce the study and gave a brief synopsis of its intent. A purposive sampling technique was employed to ensure that those with experience of the key attributes under investigation were recruited. Antenatal care nurses in each hospital assisted in initial recruitment by identifying suitable participants meeting the following inclusion criteria: Thai pregnant adolescents, aged 15–19 years, experienciing their first pregnancy at any stage of pregnancy prior to delivery, and willingness to share their experiences with the researcher. Prospective particinants were given an information sheet and consent form for themselves and parents or guardians to sign.

Eligible participants who agreed to take part in the study were asked to complete a contact detail slip and to place it in a collection box for the researcher to retrieve it from a secure location in the antenatal care clinic. The researcher collected the contact detail slips on a daily basis and contacted participants 24 hours later by phone to ask screening questions again, to verify the inclusion criteria, confirm their willingness to participate and to arrange the interview. If they declined to take part in the study, no further contact was made.

Among 25 potential participants from the first setting study, 12 replied, of whom two later withdrew. 10 participants were ientified from both the second and third study settings. Potential participants were reminded to complete the consent form for participants and for parents/guardians to take part in the interviews. The researcher conducted interviews at a time preferred by participants after accessing the antenatal care service for their next clinical appointment.

We conducted in-depth interviews with 30 eligible participants, face to face in a private room at the antenatal care services. Interviews were conducted in the local language and audio-recorded.

### Data Analysis

The ATLAS.ti software programme was used to assist in organising the large volumes of data and for the coding process. The phenomenological interpretation in this study followed the steps initially identified by Manen^16^ and Smith, Flowers and Larkin.^17^

Data were analysed through intensive reading and re-reading to obtain a comprehensive understanding of data content and context. The lead researcher began the process of identifying the meaning units for each interview. The texts were then scrutinised to examine what each participant was saying about the phenomena and specific attitudes and experiences were assigned a code. Next, all the coded units about particular phenomenon that appeared to be similar were grouped and developed to become sub-themes. This process allowed the identificiation of links between sub-themes and the subsequent identification of boader themes which in turn provided an organised interpretation of participants’ experiences.

### Ethical approval

The protocol for this study was reviewed and approved by the Faculty of Medicine and Health Sciences Research Ethics Committees, University of Nottingham (UK) and Human Ethics Committee, University of Phayao (Thailand), prior to any data collection.

## Results

### Participants’ Characteristics

The participants in this study comprised 30 adolescents, aged between 15 and 19 years at the time of interview and experiencing their first pregnancy; 17 were aged 15-16 years whilst 13 were 17-19 years old; 15 were in the second trimester of pregnancy (14–26 weeks), 9 were in the third trimester of pregnancy (27-40 weeks) and 6 were in the first trimester of pregnancy (1-13 weeks) at the time of interview.

Two participants were single and 14 were married, 14 lived with their partners and were unmarried. 15 were studying, either formally or informally. Ten participants were in work, whilst 5 were unemployed. Details are shown in Table 1:

**Table 1.** Participants’ Characteristics.

	Characteristics	Number of Participants
Age	15–16 years	17
	17–19 years	13
Stages of pregnancy	First trimester (1–13 weeks)	6
	Second trimester (14–26 weeks)	15
	Third trimester (27–40 weeks)	9
Marital status	Cohabiting	14
	Married	14
	Single	2
Occupation	Student	15
	Employed	10
	Unemployed	5

### Experience of Sex and Contraception Among Pregnant Adolescents

Five themes were identified, and reflected the phenomenological interpretation of participants’ experiences of ‘*Premarital cohabitation and sex’*, ‘*Staying in the relationship*’, ‘*Unforeseen future*’, ‘*Parental conformity*’, and ‘*Male command*’. These themes illustrated accounts of sex and contraception and appeared throughout the narrated experiences associated with adolescent pregnancy, manifesting researcher and participant co-creation in new ways of understanding the researched phenomena.

## Premarital Cohabitation and Sex

Adolescent premarital cohabitation naturally increases the possibility and probability of sexual intercourse occurring, thus increasing the chances of adolescent pregnancy. Premarital sex is a source of stigma in many regions and can negatively affect adolescent girls’ sexual and reproductive health. A large proportion of participants recalled being sexually active during cohabiting and reflected that most adolescent pregnancy occurred outside of marriage. The following participant was actually in a cohabiting relationship before becoming pregnant, but she was not consciously aware of the connection between cohabitation and pregnancy.

I lived with my partner at that time. We were students. We had unprotected sex… when I lived in my partner’s house; we didn’t use any method of contraception. Well, after we had been dating for a year, we moved in together to live at his place. [KH11; 5:72]

### Unexpected Events

Adolescents appeared not to always fully consider the consequences of their actions. They were therefore faced with the results of unprotected sex, especially unplanned pregnancy. Having sex without protection can also lead to the risks of unintended/unplanned pregnancy, sexually transmitted infections (STIs), encompassing HIV/AIDS. Some participants described the surprise of pregnancy as an unexpected event because they had assured themselves that they would not be pregnant.

I didn’t think it happen with me. We (she and her partner) never talked about marriage or having a baby. We were just in a relationship and lived together. [SH02; 15:24]

The most common misconception among many participants was related to ‘*not thinking*’ that they needed to use contraception, and their belief that pregnancy would not happen to them.

Most of the participants’ stories appeared to show a negotiation in progress between the development from adolescent or student to adult. This conflict in development had negative aspects as the following narrative demonstrates:

I had never thought about this before. When it came to me, I thought I was not a good person. I was not ready because I had not finished my school yet [PH04; 9:22]

### Disappointment

The feeling of disappointment pertained to participants feeling they had not met what they expected of themselves and others expected of them. Participants revealed disappointment due to failing to fulfil their parents’ aspirations for them to pursue further higher education and careers:

I felt I disappointed her [her mother]. That’s why I decided to keep it as a secret. There is no secret in this world! My tummy was growing. When I finally told my mum about the pregnancy, she was really disappointed in me. She asked me why I did not use contraception [PH14; 11:14]

Participants’ stories revealed feelings of guilt due to disappointing parents and the fear that the pregnancy would not be accepted by others. Some participants expressed that they learnt moral life lessons from their experiences.

I felt I made a mistake. I apologized to her [her mother] first and I then told to her later. I called her and said that “I’m sorry, I disappointed you” [ KH07; 7: 9]

## Staying in the Relationship

Intimacy in the relationship between male and female adolescents leads to sexual activity and thus possible pregnancy. Adolescents believed that romantic relationships and having premarital sex could help to maintain their relationships with their partners. Tey cohabited with their partners in close relationships in order to keep their relationships strong.

*I had the relationship and lived together with my partner. My parents knew about this. I need to keep relationship with my partner as long as possible. However, we didn’t plan to do anything and didn’t plan to have a wedding, including not planning to use contraception. It should be protected but I didn’t think about it. If I was aware of it and prevented from being pregnant, it might not have happened.* [PH01; 22:79]

### Emotional Dependence

Emotional dependence refers to a state where one partner’s self-worth becomes tied into the actions and attention of another. Many female adolescents become emotionally dependent on their partners due to an underlying fear of losing them and as such, allowed the wishes of their partner to dominate their own. They also felt locked in a cycle of emotional dependency and believed that they could not manage by themselves and were terrified of being abandoned:

I was worried. I was afraid that he wouldn’t take responsibility. He treated me very well before I was pregnant. Then, he became distant. [KH11; 5:14]

When my partner and I had a sexual relationship, we didn’t use any birth control methods. This is because my partner didn’t let me take it. He said if I took contraception pills, he wouldn’t let me live with him. I feared him getting mad. So, I didn't use any contraceptive methods" [SH09; 21: 6]

## Unforeseen Future

Increasing risk of early pregnancy among adolescents was associated with misconceptions about contraception in terms of never using contraception or using it inconsistently or incorrectly.

### Non-Use of Contraception

The majority of adolescent who participated in this study (who were pregnant and by no means representative of Thai adolescents in general) did not use contraception, and they did not think that they would become pregnant. This was because of their experience of not getting pregnant instantly when they cohabited and had unprotected sex with their partners previously. One participant who had an unintended pregnancy gave a reason for why she was pregnant:

I didn’t think that I would be pregnant. We never used any contraceptive methods when we had sex and I never got pregnant. So, I thought it would be fine. [PH14; 11:16]

### Inconsistent or incorrect Use of Contraception

Using the pill inconsistently was common due to forgetfulness, which caused unintended pregnancies among some adolescents:

I sometimes take contraceptive pills. I think it might not have an effect, but it failed. I took contraceptive pills some days and I didn’t take them [on other days]. I think it can protect for a long time. I take it 3 days per time. [KH07; 7:26]

The narratives from the participants showed that some of them had negative attitudes toward contraception, including a dislike for birth control pills and their side effects, of which they cited headaches, nausea, weight gain and moodiness. Consequently, the fear of such side effects led to contraceptive failure in some cases, resulting in unplanned adolescent pregnancy.

I used pills but I was allergic and faced with bad side effects so that I didn’t use them. When we had sex, we used natural birth control for about a year. We then didn’t use any birth control methods [SH10; 30:42]

## Parental Conformity

In traditional Thai society, cohabitation and premarital sex are met with social disapprobation and dishonour, but such social mores and shame culture are increasingly receding across all socio-economic strata.^18,19^ In many families, parents assent to their adolescent children cohabiting prior to marriage, and having sexual relationships. Adolescent cohabitation and intimate sexual relationships naturally increase the possibility and probability of adolescent pregnancy during school age. One participant described that her own parents and those of her partner allowed the unmarried adolescent couple to live together:

My partner and I became lovers about 1 year before I got pregnant. My family and his family knew that we were having a sexual relationship. They agreed to our relationship. So, I stayed with his family a few weeks and then we stayed with my family the rest of the time. [PH14; 11: 5]

### Inadequate Parent–Adolescent Discussions

Having early sex and romantic relationships without adequate parent-adolescent discussions can lead to unplanned adolescent pregnancy and risk of sexually transmitted infections. Examples of discussions reported in the data tended to have a strong focus on abstinence until marriage or until completing school, and contraceptive use, as the most effective way to avoid the dangers associated with sex. In line with that, the lack of information on how to prevent pregnancies emerged from the data as one of the main reasons for unintended pregnancy among adolescents.

The participants pointed out that their parents failed to inform them about how to prevent pregnancy or for many, the information was too little and too late as they were already pregnant.

My mom had a child when she’s 16 years old. When she knew I got pregnant, she said I shouldn’t have the child at that time and I should have teenage living. However, she didn’t prohibit me to have a relationship with my partner and we never talked about protecting from pregnancy with any contraceptive methods. She might have been fearful that this would be accepting my having sexual intercourse. But my grandparents prohibited me because they were afraid I was the same as my mom. [SH04; 20:41]

## Male Command

Female adolescents faced power imbalances in their relationships with their partners, including a lack of negotiating power concerning contraceptive use. As a result, the adolescents avoided using contraception, even when they had utilised them previously. This highlighted that the decisions of contraceptive use among adolescents remain the prerogative of males. One participant reflected that she obeyed her partner without question, and was uncomfortable to discuss the issue of contraception with him:

I took birth control pills. My mum brought the pills to me… When I lived at my partner’s house, we didn’t use any method of contraception. He told me “Don’t take the pills”, so I didn’t take them. I missed my period soon after. [SH03; 7:24]

## Discussion

### Cohabitation and Sexual Relationship

Having premarital cohabitation and sex in early young age has been shown to be a crucial cause of girls becoming pregnant and is linked to increases in non-marital fertility. Although this evidence has become an increasing common part of life for adolescents, in some regions in Thailand it remains stigmatised and shameful for the family and in society. This can be seen in several research studies in some rural regions in Thailand and elsewhere. Neamsakul’s^19^ and Ounjit’s^18^ work in rural areas determined that cohabitation before marriage is still disapproved of for either adolescents or adults, because it is considered disgraceful and in contradiction to Thai tradition. Interestingly, there is evidence that male adolescents generally see cohabitation before marriage as normal, whilst female adolescents see marriage as being important.^20^ However, adolescents in this study were willing to live together and engaged in sexual relationships with their partners in order to contine the relationship. They exhibited an increasing acceptance of premarital sex with their partners they love. This is in a line with a study of Manning and Cohen^21^ which reported that in the American family, there has been dramatic increase in cohabitation and growth in non-marital fertility in recent decades. They also found that cohabitation was the most common American family formation during adolescence and that the most pregnant adolescents developed a cohabiting union before the birth of their children.

With the increasing acceptance and prevalence of adolescent premarital sexual activity, most participants in this study were unprepared for becoming pregnant and parenthood. Moreover, they and their partners were not always consciously aware of the connection between cohabitation, sexual relations and pregnancy. The desire of closeness drove the search for romantic relationships with partners. Therefore, the adolescents were willing to give everything to their partners.^22^ As noted by Azmawaty,^23^ the stage of adolescent life in general is associated with risk-taking behaviour and lifestyle experimentation, including, for some, pre-marital sexual experimentation 

### Family: Conformity

Little is known about the association between parental approval and adolescent sexual behaviour, which appears to be becoming more prevalent in rural areas in Thailand. Various parental dimensions (including connectedness, material support, behaviour monitoring, and parent–child communication) are expected to mediate adolescent behaviour, including classifying, screening, and providing information for the socialisation of children and adolescents.^24^ The trend of living together before marriage has become normal for many families. This reflects changing Thai family patterns.^25,26^ Adolescents are more likely to engage in risk-taking behaviour, including in sexual activity, and parental acceptance of such behaviour was manifest in some participants’ accounts (e.g. overt approval of cohabiting with sexual partners).^26^ A constructive parental role regarding adolescent sexual and reproductive health is associated with reduced levels of risk-taking behaviour in their children. Parental monitoring, discipline and communication with fathers, for female adolescents, can predict transition to first sexual intercourse and has been shown to delay having premarital sex and increase contraceptive use.^27^ It has also rich potential as an intervention to impact positive adolescent’s sexual decision-making and contraception use.

A longitudinal study in Scotland showed that low parental monitoring was associated with an earlier transition to sexual activity for both females and males, and also a greater risk of unprotected sex for females.^28^ In this sense, parental approval is important in daughters’ activities, and an evaluative response to how adolescents are doing. However, Okigbo, Kabiru, Mumah, Mojola and Beguy^27^ studied African adolescents in Kenya and argued that parental monitoring was not associated with transition to first sexual intercourse. It is important to consider that interventions for parents should focus on enhancing their ability to share their values and guide their children’s behaviour as well as maintain open communication about sexual and sensitive issues. Prospective reciprocal relationships were found between parental monitoring and sexual risk behaviour for male adolescents, and between perceived peer risk involvement and sexual risk behaviour for female adolescents.^29^ Therefore, promoting health should be driven by a campaign for adolescents to raise awareness of the consequences of sexual relationships without contraception use.

Providing sex education is an opportunity to encourage family values in children. Open communication about sex between parents and adolescents is a crucial. Notwithstanding, the core values in Thai society restrict the discussion of sex education issues. It can be seen in this study that the participants did not always discuss sex and contraception use with their parents. This was due to feeling uncomfortable talking about sexual issues. Sridawruang^30^ also revealed that the most Thai parents failed to provide sex education for their children which resulted in missed opportunities to impart knowledge about sexual issues, health and well-being. The limited provision of sex education by Thai families will impact on the extent of control over what and how adolescents learn about sex. This is in a line with a study by Ashcraft and Murray^31^ who pointed out that communication about sexuality between parents and their children can create anxiety and apprehension and may lead to avoidance of discussions. In fact, sex education and parent-children communication about sexuality are associated with delayed sexual relationships and more consistent use of contraception.^32^ Hence, parents should be the first source of information about sex for their adolescents. Understanding correct information can protect children from risky behaviours as they grow up. Additionally, interventions promoting parental communication with adolescents about sexual relationships have the potential to empower parents to have adequate knowledge and skills to nurture the sexual reproductive health of their growing adolescents.

### Adolescent Women: Emotional Dependence

The unique role of romantic relationships influences the expression of sexual behaviour within relationships. Adolescents in this study revealed that they felt having premarital sex and living together with their partners led to intimate relationships and influenced future relationship goals. Having unprotected premarital sex and agreement with their partners’ decisions were negotiation tools to maintain and sustain relationships. This is in line with a study of Pogoy, Verzosa, Coming and Agustino^22^ which found that adolescents can be willing to make significant compromises for their partners in exchange for closeness and security. The adolescents were also more likely to have sexual relationships when they felt a sense of intimacy in their partnership.^33^

### Adolescent Men: Male Command

The majority of participants never used any contraceptive methods for pregnancy and STI prevention or used them inconsistently. There were large gender-based differences in sexual conduct and in the ability to negotiate sexual activity and contraception use.^34^ Gender norms preserved adolescents’ ignorance about reproductive health and shared responsibilities in a relationship. This can be seen in female adolescents being expected to take responsibility for pregnancy prevention, but not giving them encouragement or facilitation to seek reproductive health information and services. Notwithstanding, male adolescents also lack encouragement to take responsibility for the prevention of pregnancy.^35^ Congruent with previous studies, findings from Nigeria by Olley,^36^ exposed that engaging in sexual practices reduces female partners’ bargaining power to insist on the use of barrier contraception or insisting on safe sex. Gender norms can also put males in a position of sexual dominance and limit females’ ability to control their own reproductive health.^37^ As can be seen other studies showing males resisting using contraception and their female partners lacking power to negotiate its use.^37,38^ Female adolescents have also been shown to have been pressured by their partners into decision-making to waive contraception use.^38^

There is widespread resistance to using condoms in long-term relationships due to the implications of a lack of trust and dishonesty in sexual relationships. Eggers, Aarø, Bos, Mathews, Kaaya, Onya and de Vries^39^ studied 3 Sub-Saharan African countries and found that in traditional gender roles, it was assumed that men would practice safe sex by taking the initiative and responsibility for buying condoms and negotiating sexual behaviours. However, Nalukwago, Crutzen, van den Borne, Bukuluki, Bufumbo, Burke, Field, Zikusooka, Fiedler and Alaii^40^ argued that behavioural intention to use condoms and self-efficacy to use them were not linked to gender norms of either male or female adolescents in sexual practice.

Therefore, the concept of gender norms should be addressed with regard to information about adolescents’ reproductive health, in order to change negative gender norms that hinder condom use and the use of other contraception. The need to strengthen family planning also enables behavioural change among men, to promote gender and reproductive health rights and empower women with better negotiation skills. Data driven, an information campaign adressing negative gender norms should be considered. Information and health communication services, such as mass media and interpersonal communication, should be used to address gender norm-based misconceptions.

### The Power of Inclusive Sex Education and Contraception

Young adolescents often lack knowledge about the implications of sex relationship and contraception use, which can result in their becoming pregnant. Gyesaw and Ankomah^41^ and Van Zyl, van Der Merwe and Chigeza^42^ showed that adolescents were curious about sexuality but they lacked sexual education. Likewise, this study illustrated that most of the female adolescents never used any contraceptive methods or used them inconsistently and highlighted risky sexual practices that result in them becoming pregnant. Previous studies in Thailand found that Thai adolescents’ inappropriate knowledge about safe sex and ineffective contraception use resulted in increased adolescent pregnancy,^43^ along with feeling uncomfortable using condoms, and a lack of acceptance of condom use on the part of the sexual partner.^44^

The main barriers to modern contraception, especially pill uptake among adolescent women in this study were misconceptions and fear of side effects, which led to contraceptive failure in some cases. These side effects of contraceptive pills (e.g. nausea, headache and migraine, weight gain, breast tenderness or mood swings) were barriers to use and this supports findings from previous studies.^26,45,46^ This study also revealed that the reasons for discontinuation or non-use of family planning were lack of sex education and contraceptive methods as well as fear and concern about contraception. Meanwhile, other studies illustrated a similar barrier to contraceptive use in terms of fear of side effects by users. A study in Kenya found that peers and other community members were the main sources of information on contraception rather than health care providers and other educational sources, and this heavily influenced the decision whether or not to use contraception.^46^

Having premarital sex at an early age is often associated with unsafe sex, in part through lack of knowledge, skills, and self-efficacy to negotiate contraception This does not occur in a vacuum: a myriad of interconnected factors are involved at the individual, family, community and national levels, including education, religion, deprivation, inequality and ethnicity. As young people move in and out of romantic relationships, one might expect their use of contraception to fluctuate with these transitions. This increases the risk of gaps in protection and unprotected sex, as well as contraceptive switching. Sex education provides an important platform for providing information on using contraception and should be recommended to designed for adolescents. Promoting healthy adolescent sexuality is not only a responsibility for teachers, schools or health care providers, but also to be a collective responsibility of parents. Educating parents and their adolescents regarding the most effective methods may increase compliance and adherence.

## Conclusion

The vast majority of first sexual experiences in adolescents happen in the context of romantic relationships that tend to involve conflicts or tensions between their parents and their romantic partners, often compelling the adolescents to choose. Although the issue of sex outside of marriage hostorically became a significant mortal sin in both Western and Thai societies, in some areas parents now exhibit general willingness for their unwed adolescent daughters to cohabit with their male partners, facilitating premarital sex, sometimes resulting in adolescent pregnancy. This was not only a major shift in Thai culture, but it was also in Western (Christian) cultures, where the traditional importance of the sacrament of marriage was particularly significant. Attitudes have changed and cohabitation and premarital sex are now widely accepted, reflecting changes in socio-cultural context. Conversely, parents often believe that strict supervision of adolescents inhibits their establishment of romantic relationship and premarital sex, but this is not necessarily the case.

Additionally, parent–child communication about sexual issues and contraception use is averted when parents feel uncomfortable and fear as they would perpetuate their own early sexual practices and negative attitudes towards contraception.^47,48^ Therefore, given the potential that parent-child communication about sexual issues may have in reducing adolescent sexual risk,^47,49^ the findings suggest the efforts to increase parent-child communication may benefit from using open communication with neutral messages and appearing comfortable to display positive attitudes towards communication around sex and contraceptive use.

A potential source of social and emotional support for adolescents is a positive relationship with their parents. Parental closeness has been associated with reduced adolescent pregnancy, sexual behaviour and early sexual debut.^50^ Although previous studies found that pregnant adolescents tended to hide their pregnancies for as long as possible due to their parents’ discomfort and disapproval of sex, other parents who were more admiring and accepting of their daughters’ sexual practice may be more permissive.^19,51^ However, greater parental permissiveness has also been associated with increased risk of adolescent behaviours, including more frequent sexual practices and an elevated risk of pregnancy.^52^

Every adolescence involves life-changing decisions about sexual and reproductive health. The lack of sexuality education to inform decisions causes adolescents to be faced with unintended adolescent pregnancy and leaves them vulnerable to coercion. Lack of awareness of contraceptive use is also strongly associated with increased risk of early pregnancy. Disadvantaged adolescents are more inconsistent in their use of contraception, even when they do not want a pregnancy, and they tend to have more unintended pregnancies.

There has been a growing interest in patterns of contraceptive use among adolescents due to the social relevance attached to pregnancy in this age group. Negative attitudes towards contraceptive methods and fear of the side effects of pills as well as adverse reactions are a major barrier to use. The effect of education on attitudes towards contraceptive use have been established.^26^ A study in Thailand^53^ found that adolescent women who achieve well in school and do not exhibit risk behaviours are more likely to become pregnant when they do not have sufficient knowledge and skills to protect themselves from risky sexual behaviours, and that adolescent men did not understand how contraceptive methods worked.
